# Bioenergy potential of agricultural straw residues from Southern Spain

**DOI:** 10.1038/s41598-026-46840-z

**Published:** 2026-04-21

**Authors:** M. Guadalupe Pinna-Hernández, Rubén López Pastor, Manuel J. Díaz Villanueva, José Luis Casas López, Francisco Gabriel Acien Fernández

**Affiliations:** 1 Department of Chemical Engineering, University of Almería, Carretera de Sacramento s/n, 04120 La Canada de San Urbano, Almería Spain; 2 Solar Energy Research Centre (CIESOL), Joint Centre University of Almería- CIEMAT, 04120 Almería, Spain; 3 Department of Chemical Engineering and Food Technology, Wine and Agrifood Research Institute (IVAGRO), University of Cádiz - International Campus of Excellence (ceiA3), 11510 Puerto Real, Cádiz Spain

**Keywords:** Biomass, Net calorific value, Fusibility, Circular economy, Sustainability, Renewable energy, Environmental sciences, Plant sciences

## Abstract

This study presents a comprehensive and harmonized assessment of eight agricultural straw residues: barley, corn, oatmeal, rapeseed, rice, sunflower, triticale, and wheat. Sourced from Southern Spain (Andalusia), these residues were assessed through 75 physicochemical and energetic analyses performed under standardized CEN/ISO methodologies. The resulting dataset allows a robust evaluation of their suitability as solid biofuels for thermochemical energy conversion, supporting strategies to reduce CO₂ emissions and promote circular economy practices in Mediterranean agricultural regions. Most straws exhibited low ash contents (5.03–7.44%), meeting ISO 17225 quality classes A or B, whereas rice straw showed distinctly higher values (15.28%). Volatile matter ranged between 75 and 80% for all species except rice (70.09%). Net calorific values (15.0–19.0 MJ kg^−1^) were comparable to those of typical herbaceous biomasses, confirming the strong energy potential of these residues. Elemental analysis showed adequate carbon contents for combustion (45.70–49.73%), except for rice straw (41.10%). Nitrogen levels were consistently below 1.5% w/w, meeting Class A limits for NO_x_ emissions. Sulphur concentrations remained within 0.20–0.30% w/w for most species, although rapeseed and corn straw exhibited elevated levels (0.65% and 0.30%, respectively). Empirical linear correlations were developed to predict the net calorific value from ash, volatile matter, carbon, hydrogen, and chlorine content, with species-specific models achieving high predictive performance. These correlations provide a practical tool for rapid energy quality estimation and process design. Overall, the study confirms that agricultural straw residues represent a promising renewable feedstock for heat generation within regional bioenergy systems.

**Supplementary Information:**

The online version contains supplementary material available at 10.1038/s41598-026-46840-z.

## Introduction

One of the current global issues is the energy crisis. On the one hand, we are facing the depletion of fossil fuel resources, and on the other, the contribution their use makes to climate change through the release of greenhouse gases into the atmosphere. In recent years, the European Union has expressed concerns regarding the use of nuclear energy as an alternative to fossil fuels, as well as the implementation of stricter regulations on greenhouse gas emissions, particularly carbon dioxide (CO₂), methane (CH₄), and nitrous oxide (N₂O) and the renewable energy consumption fraction had been increased up to 32% for 2030. In this context, extensive research has been conducted on renewable energy sources such as hydraulic, wind, solar, geothermal, and biomass energy^1–9^. Biomass is expected to play a pivotal role in the global energy system. Projections indicate that by 2050, approximately 15% of the world’s primary energy supply will be derived from biomass resources^10,11^. Biomass is defined as non-fossilized organic matter of biological origin, and it is considered the fourth largest source of biofuel-based energy after coal, oil, and natural gas.

One of the main advantages of using plant-based biomass is its potential to reduce net greenhouse gas emissions compared to fossil fuels, as the CO₂ released during combustion can be partially offset by the CO₂ absorbed during biomass growth; however, the overall climate impact depends on life-cycle factors such as agricultural practices, land use, processing, transport, and conversion efficiency. This makes it a much more sustainable and environmentally friendly option compared to the main energy sources currently in use^3,12,13^. Another advantage of using plant-based biomass as an energy resource is its availability. Although biomass can be obtained from woody materials such as pine, poplar or eucalyptus, the possibility of using agricultural residues as an energy source is also being explored. In the case of extensive agriculture, it occupies approximately 4,781 million hectares worldwide. Within Europe, France stands out with around 12.7 million hectares, while Spain accounts for approximately 17 million hectares^14–16^. Straw crops represent a significant share of agricultural production. In 2023, the combined area dedicated to Barley, Cereals, Corn, Mixed Grain, Oats, Rapeseed, Rice, Sunflower, Triticale, and Wheat amounted to 6.24 million hectares, yielding a total production of approximately 12.8 million tons. Among these, Barley and Wheat stand out, with cultivated areas of 2.3 million hectares and 1.9 million hectares, respectively, and production volumes of 3.7 million tons and 4 million tons (Table S1). In Andalusia, we can also find the presence of several of these crops. In 2024, the cultivated area of barley covered 105,385 hectares, yielding 235,409 tons. Mixed grains accounted for 35,641 hectares, while oats occupied 95,177 hectares with a production of 175,742 tons. Rice was cultivated on 37,302 hectares, wheat on 295,624 hectares with a yield of 1,003,594 tons, and maize on 4,862 hectares, producing 55,229 tons.^17,18^. These data highlight the potential value of utilizing residual biomass derived from the agricultural activity of these crops in Andalusia.

This large amount of biomass represents a vast amount of potential energy resources that are currently not being utilized and are instead discarded as waste. Therefore, it is of great interest to revalorize these residues and to study which types of plant waste are suitable for use as an energy source, since these residues can account for up to 75% of the plant material, especially during the summer months, when the long growing season comes to an end^19^. This would promote the circular economy and improve the sustainability of the agricultural industry. However, the processing of agricultural plant residues is a labor-intensive procedure due to their heterogeneity and seasonal nature. Additionally, several factors must be considered that hinder the combustion of this resource, either because of a low calorific value, which renders it unviable due to its composition (with carbon content and moisture being commonly associated parameters), or because it is unsuitable for combustion processes due to the emission of harmful gases, such as those resulting from high concentrations of sulphur, nitrogen oxides, or chlorine. Furthermore, poor ash fusibility may also make it incompatible with boiler combustion. For all these reasons, a prior characterization of the crop residues intended for energy use is required^12,19,20^. When analyzing biomass, standardized official methods must be followed, such as those established by the American Standard Testing Methods (ASTM) and the European Committee for Standardization (CEN), or those developed by the Technical Committee ISO/TC 238. Typically, the chemical analysis of biofuels includes major components such as total carbon (C) and hydrogen (H), sulphur (S), chlorine (Cl), and nitrogen (N), as well as other elements including Al, Ca, Fe, K, Mg, Na, P, Si, and Ti, and trace elements such as As, Ba, Cd, Co, Cr, Cu, Hg, Mn, Mo, Ni, Pb, Sb, Tl, V, and Zn. In addition, moisture, ash content, volatile matter, gross calorific value, and net calorific value are also evaluated, along with ash fusibility characteristics^3,12,19–22^.

In this context, the objective of this study is to identify and evaluate the main physicochemical parameters governing the suitability of different agricultural straws as solid biofuels under real and standardized conditions. Beyond individual characterization, the study addresses a relevant scientific gap by providing a statistically robust and harmonized comparison of eight straw types: six cereals (barley, corn, oats, rice, triticale and wheat) and two oilseed crops (rapeseed and sunflower), based on 75 independent samples collected within a single, well-defined Mediterranean region (Andalusia, Southern Spain). All materials were analyzed using standardized CEN/ISO methodologies, enabling direct comparability among feedstocks and reducing methodological bias commonly found in literature-compiled datasets. In addition, net calorific value (NCV)–oriented linear correlations were developed to link energy performance with proximate and ultimate composition, providing practical tools for rapid energy-quality screening. Notably, this work contributes original and consolidated data for rapeseed and triticale straws, which remain poorly represented in previous bioenergy studies despite their increasing agricultural relevance.

## Materials and methods

### Samples and origin

In this study, 8 different types of straw have been analysed, provided by local farmers from the Andalusia region (Southern Spain), from different crops of: rapeseed (*Brassica napus*) (RA), oatmeal (*Avena sativa*) (OA), triticale (*Triticum aestivum*) (TR), rice (*Oryza sativa*) (RI), corn (*Zea mays*) (CO), barley (*Hordeum vulgare*) (BA), sunflower (*Helianthus annuus*) (SU) and wheat (*Triticum vulgare*) (WH). Thus, a total of 75 samples were obtained from a variety of sources: biomass production companies, agro-industry or wood transforming industry biomass wastes, and agricultural cooperatives throughout the Andalucía region (Southern Spain). The origins of each collected straw (Seville, Cordoba and Jaén) and the number of individual samples analysed for each type are shown in Table S2.

### Analysis methodology, preparation, and moisture content

Biomass samples were prepared and analyzed according to the methodology developed by Technical Committee ISO/TC 238 in collaboration with European Technical Committee ENC/TC 335 Solid Biofuels^23^.

In this study, each analytical determination was performed in technical triplicate, consisting of repeated measurements of the same prepared sample to assess analytical precision; the resulting mean value was used for all subsequent statistical analyses (ANOVA, PCA and regression), while the triplicates were not treated as independent samples.

The methodology used for preparation of different straws collected in fields until achieving the analysis samples was carried out in accordance with (ISO 14780:2017^25^). Thus, the biomass samples received by local farmers are left uncovered for one week until their moisture content is reduced to 20%. Then, they are milled using a cutting mill (Retsch SM2000) to obtain particles between 5 and 10 mm. Subsequently, the particle size is further reduced to below 0.25 mm through a second milling process using an ultra-centrifugal mill (Retsch ZM200). According to this methodology biomass test portion for analysis must be thoroughly mixed and reasonably stabilised under laboratory temperature and humidity conditions. Thus, the samples with a particle size below 0.25 mm were stabilized in laboratory for 24 h, and they were stored in plastic flasks with a tight-fitting lid. According to the standard methodology followed for each analysis (described below), in all cases, 1 g prepared analysis samples were used to determine their moisture content ${M}_{ad}$ under controlled moisture and weight conditions (ISO 18134-3:2024^26^), which allows all results to be obtained on a dry basis^27,28^.

### Ash content and fusibility

The ash content was determined following the ISO 18122:2022^29^ methodology. Firstly, ceramic crucibles capable of withstanding high temperatures were preheated at 550 °C for at least 1 h to remove any possible impurities or dirt. After cooling, the crucibles were weighed (m_1_) and then filled with the sample (m_2_). The samples were heated in a muffle furnace (Nabertherm LVT 15/11), with the temperature gradually increased to 250 °C for 30–50 min at a heating rate of 4.5 °C/min to 7.5°C/min. Once 250 °C was reached, the temperature was maintained for 60 min to allow volatile compounds to evaporate before ignition. Subsequently, the temperature was further increased to (550 ± 10) °C at a ramp rate of 10 °C/min and maintained for 120 min. Finally, the crucibles containing the generated ashes were cooled in a desiccator and weighed (m_3_). The dry base ash content, ${A}_{d}$, of the sample expressed as a percentage of mass on a dry basis should be calculated with (1):

1 $${\mathrm{A}}_{{\mathrm{d}}} = \frac{{{\mathrm{m}}_{3} - {\text{ m}}_{1} }}{{{\mathrm{m}}_{2} - {\mathrm{m}}_{1} }}{ } \times { }100{ } \times \frac{100}{{100 - {\mathrm{M}}_{{{\mathrm{ad}}}} }}$$

The ash fusibility of the studied samples was estimated following the international standard procedures for hard coal and coke (ISO 540:2008)^30^ using imaging sintering point testing equipment (Leco AF-700). First, enough ash samples were obtained through combustion in a muffle furnace at 550 °C, as described previously. Once prepared, the ash samples were milled in an agate mortar and sieved to ensure uniform size and shape. Subsequently, with the assistance of an iron mould, deionized water, and a dextrin solution (100 g L^-1^), the ashes were formed into a standardized pyramidal pellet. Ash fusion temperature measurements were conducted up to a maximum temperature of 1500 °C in an oxidizing atmosphere. During testing, the initial deformation temperature (DT), softening temperature (ST), hemisphere temperature (HT), and fluid temperature (FT) were recorded based on the specific transformations of the ash pyramids. These transitions (Figure S1) were captured with an integrated digital camera and software (Leco AF-700).

### Volatile matter and elementary analysis (C, H, N, S and Cl)

Volatile matter was determined from ISO 18123,2024^31^. The residue remaining in a closed crucible, after being placed in a muffle for 7 min at the temperature of (900 ± 10) °C (Nabertherm LVT 15/11). The content of volatile matter, ${V}_{d}$, of the sample is expressed as a percentage of mass in dry basis, is calculated through (2):

2 $$V_{d} = \left[ {\frac{{100 \times \left( {m_{2} - m_{3} } \right)}}{{m_{2} - m_{1} }} - { }M_{ad} } \right] \times \left( {\frac{100}{{100 - M_{ad} }}} \right)$$

where m_1_ is the weight of the closed crucible clean and empty before the analysis; m_2_ is the weight of the closed crucible with the sample (approx. 1 g); m_3_ is the final weight of the closed crucible with the residue generated during the analysis; ${M}_{ad}$ (Table S9), is the moisture sample to analysis.

### Elemental analysis (C, H, N, S and Cl)

The elemental analysis of all samples was performed using the automatic analyser (LECO Truspec CHN 620-100-400), following the ISO 16948:2015 methodology^32^. A sample mass between 0.1 and 0.25 g was combusted at 950 °C in the presence of oxygen under conditions ensuring complete conversion to ash and gaseous combustion products, such as carbon dioxide, water vapor, elemental nitrogen and/or nitrogen oxides, sulphur oxides and oxyacids, and hydrogen halides. The combustion products were processed to ensure that any hydrogen not associated with sulphur or halide combustion products was released as water vapor. Nitrogen oxides were reduced to elemental nitrogen, and any combustion products that might interfere with subsequent gas analysis procedures were removed^33^. The mass fractions of carbon dioxide, water vapor, and nitrogen in the gas stream were then quantitatively determined using specific analyzer detectors. The results for carbon (C), hydrogen (H), and nitrogen (N) were expressed as percentages on a dry weight basis.

For the analysis of sulphur, high-temperature combustion at 1350 °C of the samples in a tubular furnace (Leco Truspec S 630-100-700) was selected as the determination method according to ISO 16994:2016^34^. The quantification of the gaseous combustion products formed was carried out in a manner like the method described above for determining the total content of carbon, hydrogen, and nitrogen. The chlorine analysis was carried out in equal than sulphur according with ISO 16994:2016^34^ methodology. First, calorimetric pump digestion (Calorimeter Parr 6300) was performed on each biomass sample, following the procedure for determining the calorific value (see "Calorific value" section). Second, the chlorine collected in the washing waters generated during combustion was quantified with silver nitrate using potentiometry in an automatic titrator (Mettler Toledo G20). The results for sulphur (S) and chlorine ($Cl$) were expressed as percentages on a dry weight basis.

### Calorific value

Calorimetry is the experimental method chosen to determine the calorific value in solid biofuels by using the ISO 18,125:2017 2018^35^ methodology. To calculate the calorific value (CV), the gross calorific value (GCV) of a solid biofuel was first determined at constant volume and a temperature of 25 °C, using an automatic calorimetric pump calibrated by the combustion of certified benzoic acid (Calorimeter Parr 6300). During the assessment, the samples were ignited using an electric firing system, raising the temperature of the water inside the vessel. The temperature rise of the water in the vessel was measured during the combustion of approximately 1 g of sample (milled to below 0.25 mm) in a pure oxygen atmosphere at 30 bars. The result obtained under these conditions was the GCV at constant volume (${q}_{v,gr, d}$), with all the water from the combustion products remaining as liquid water.

In practice, biofuels are burned at constant pressure (atmospheric), and the water is either removed as steam with the exhaust gases or condensed. In both cases, the heat released during combustion is the measured net calorific value (CV) of the fuel at constant pressure (${Q}_{p,net, d}$), which was calculated from the Eq. (3) using the GCV at constant volume and the elemental analysis. The results of the calorific value were expressed as MJ kg^-1^ on a dry weight basis.

3 $${Q}_{p,net,d}={q}_{v,gr,d }-\mathrm{212,2}\times {H}_{d }-\mathrm{0,8}\times (O-N )$$

The oxygen content was not measured directly but estimated by difference according to Eq. (4) on a dry, ash-free basis, following the standard difference method described in ISO 16993:2016 and ISO 16948:2015; consequently, the ash fraction is excluded from the calculation, and the resulting oxygen value should be interpreted as an indirect estimate.

4 $${O}_{d}=100-(C+N+H+Cl+S )$$

### Bulk density

The bulk density determination consists of weighing a test portion placed in a container of a standard size and volume, according to CTN 164 Biocombustibles Sólidos, 2015^36^. Bulk density, expressed in kg m^-3^, is calculated from the net weight per standardized volume and is accompanied by the moisture result, ${M}_{ad}$.

### Statistical methodology applied to the data

In this research, both a descriptive and a correlation study of the data obtained from the 75 samples of straw crop residues were performed using STATHGRAPHICS 19. In the descriptive study, the standard parameters (moisture, ash, bulk density, volatile, total carbon, hydrogen, nitrogen, sulphur, chloride, GCV and NCV) were analyzed to determine the representative values (mean, median, variance, standard deviation, minimum and maximum). In the correlation study, the different parameters obtained from the previous analyses were first compared with the species of straws studied using an ANOVA, establishing a 95% confidence p-value. This allows us to determine if there are significant differences in certain parameters between the different species. Following this, a Principal Component Analysis (PCA) was conducted to examine how all these parameters relate to each other, aiming to establish a linear relationship between one or more parameters and the calorific value of the biomass.

Once the variable(s) that align with the calorific value were identified, a linear model was developed. This model will be based on previous study models in case the results align with those of other researchers. The model performance has been evaluated by the coefficient of determination (R^2^) and by the following statistical parameters: Mean absolute error ($MAE$) and Mean bias error ($MBE$), which measure the differences between the experimental GCV and the predicted GCV by the corresponding model.

5 $$MAE = \frac{1}{n}\mathop \sum \limits_{i = 1}^{n} \left| {Value_{experimental} - Value_{predicted} } \right|$$

6 $$MBE = \frac{1}{n} \mathop \sum \limits_{i = 1}^{n} \left( {Value_{experimental} - Value_{predicted} } \right)$$

## Results and discussion

### Ash content and fusibility

**Fig. 1:**
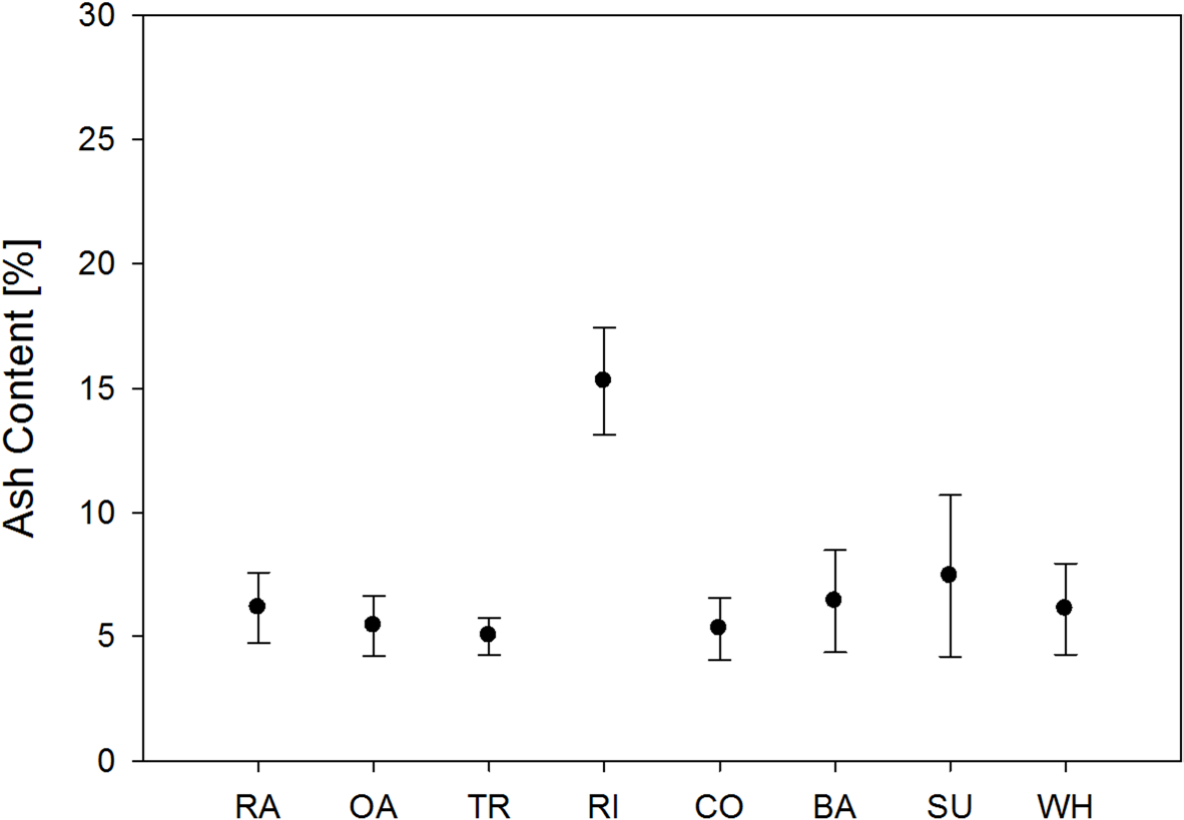
Ash content in the studied biomass samples. Rapeseed (RA), oatmeal (OA), triticale (TR), rice (RI), corn (CO), barley (BA), sunflower (SU), and wheat (WH).

When selecting the most suitable biomass for fuel, it is preferable to have low moisture content and ensure that its combustion produces minimal ash and volatile compounds. Additionally, achieving the highest possible NCV value is desirable^37^. Figure 1 shows the variability of ash content with different biomass.

Selecting biomass with low ash content is crucial, as it enhances combustion efficiency and reduces slagging, fouling and operational problems in boilers^37,38^. Among the straws analyzed in this study (Fig. 1), rice straw (RI) exhibits the highest ash content (≈15% d.b.), whereas triticale straw (TR) shows the lowest values (≈5% d.b.). These results are consistent with previous studies reporting elevated ash contents for rice-derived residues and lower inorganic fractions for cereal straws. When compared with other agricultural residues reported in the literature, whole straw fractions generally present higher ash contents than selected plant sub-fractions. For example, sunflower shells show ash contents as low as ~ 2.8% d.b. with GCV values around 17.5 MJ kg^−1^, while sunflower husks or mixed stem–floret fractions typically range between 9 and 11% ash with GCV values of 18.6–20.2 MJ kg^−1^. Similarly, corn husk and stalk fractions have been reported with ash contents of 2.4–3.6% and GCV values of approximately 16.1–16.6 MJ kg^−1^. The comparatively higher ash observed in whole straws can be attributed to their broader botanical composition, harvesting practices, and the greater likelihood of soil contamination. In this context, appropriate harvesting and handling protocols aimed at minimizing mineral contamination remain essential to improve the fuel quality of agricultural straws^39^.

Ash fusibility was also evaluated, taking into account the values of Initial Deformation Temperature (DT), which marks the ashes begin to deform; Softening Temperature (ST), where ash begins to significantly soften; Hemispherical Temperature (HT), where ash melts into a hemispherical shape; and Flow Temperature (FT), at which ash become fully liquid or stars to flow. The evaluation of ash fusibility was carried out following ISO 18122^29^, as included in the previously discussed methodology, by studying the critical temperatures at which the ashes begin to fuse. This allows for the assessment of its behavior during combustion and the risk of blockages and slag formation. These parameters are critical as they define ash behavior in the boiler during biomass combustion. It is essential to avoid researching temperatures at which ash becomes liquid and may flow through the system, as this can cause blockages and damage to the boiler.

**Fig. 2:**
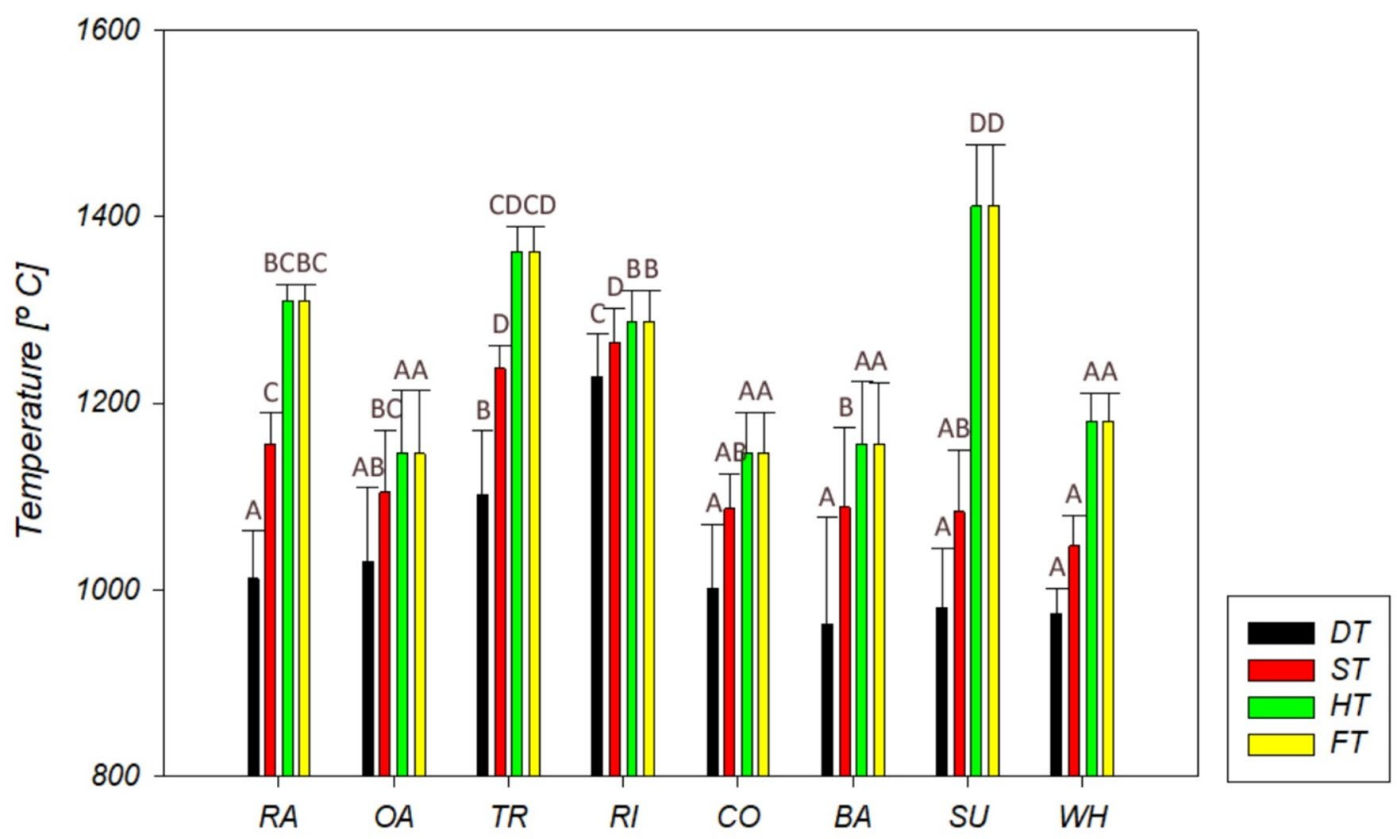
Ash fusibility in the studied biomass samples. Rapeseed (RA), oatmeal (OA), triticale (TR), rice (RI), corn (CO), barley (BA), sunflower (SU), and wheat (WH).

In this context, the most suitable biomass based on the experimental scope of this study, it is characterized by higher ash softening and melting temperatures, as determined from the measured fusibility tests; however, no direct mineralogical or elemental ash composition analyses were performed, and any discussion of underlying causes (e.g. alkali or silica effects) should therefore be regarded as qualitative and supported by literature rather than by direct evidence from this work. As shown in Fig. 2, SU straw achieves the highest combustion temperature, followed by triticale straw, with values around 1300–1400 °C. In contrast, WH, BA, CO straw, and OA straw exhibit a lower temperature range, 1100–1200 °C. These results are within a similar range (1114–1288 °C) as greenhouse agricultural residues (cucumber, tomato, and pepper)^19,22^ and for other biomass materials as Guizhou coal with a DT and ST of 1210 °C and 1311 °C, respectively or straw with a DT and ST of 1198 °C and 1257 °C^30^.

### Bulk density

**Fig. 3:**
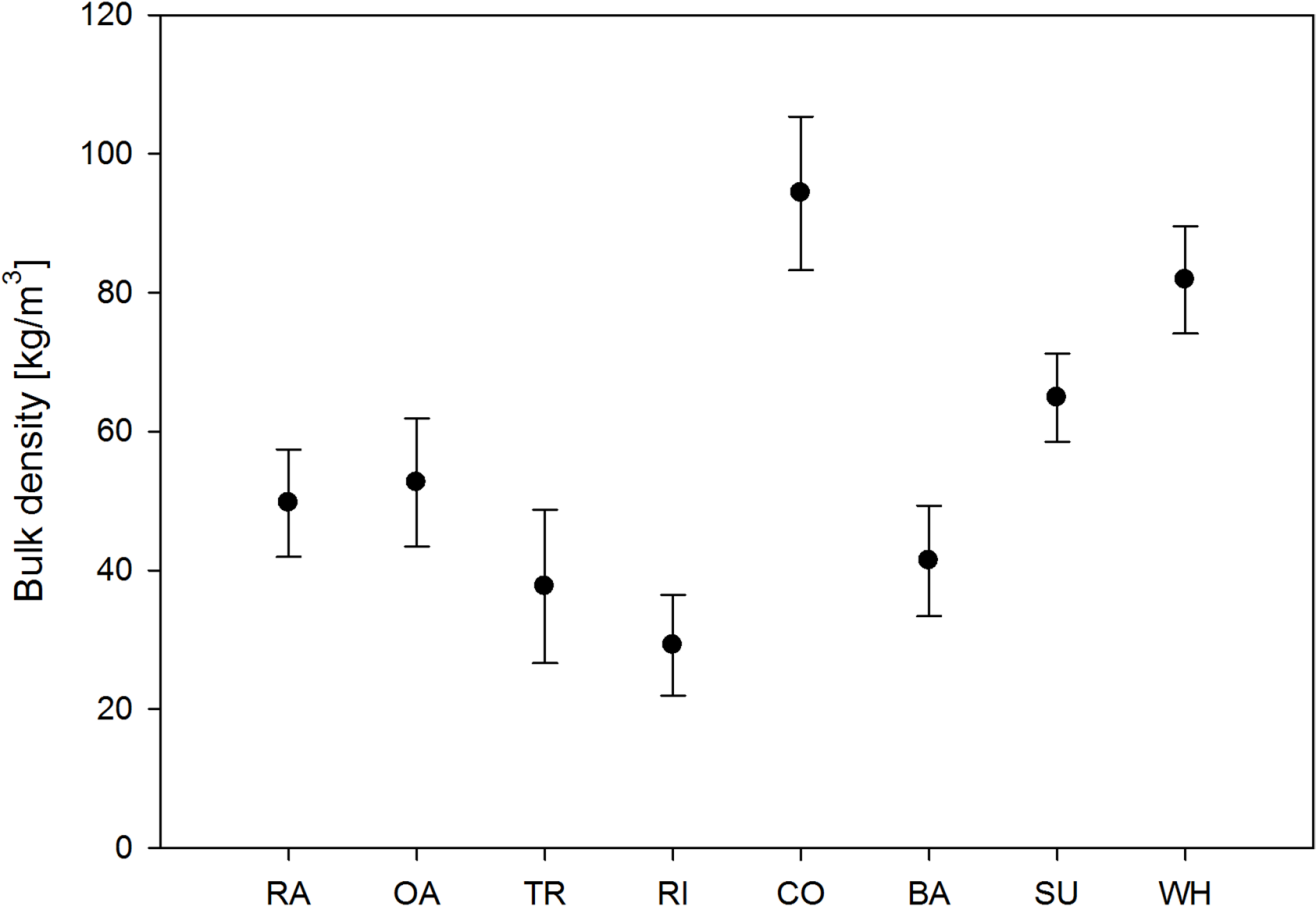
Bulk density in the studied biomass samples. Rapeseed (RA), oatmeal (OA), triticale (TR), rice (RI), corn (CO), barley (BA), sunflower (SU), and wheat (WH).

Bulk density is a key parameter for fuel supply as it allows us to relate mass to volume, thereby determining the space requirements for the transportation and storage of biofuel^40^. In this study, the measured bulk densities show clear differences among the various straw types. CO exhibits the highest bulk density (94.30 kg m^-3^), indicating that it requires significantly less storage volume per unit mass compared with the other biomasses, while RI straw presents the lowest value (29.19 kg m^-3^), implying more demanding logistical requirements. WH and SU straw also display relatively high densities (81.81 and 64.83 kg m^-3^, respectively), which could make them attractive for handling and transport. In contrast, TR and BA straw show lower densities (37.64 and 41.33 kg m^-3^), suggesting that their storage systems may need larger capacities. The associated errors remain within acceptable ranges, supporting the reliability of the measurements (Fig. 3).

### Volatile matter and calorific value

**Fig. 4:**
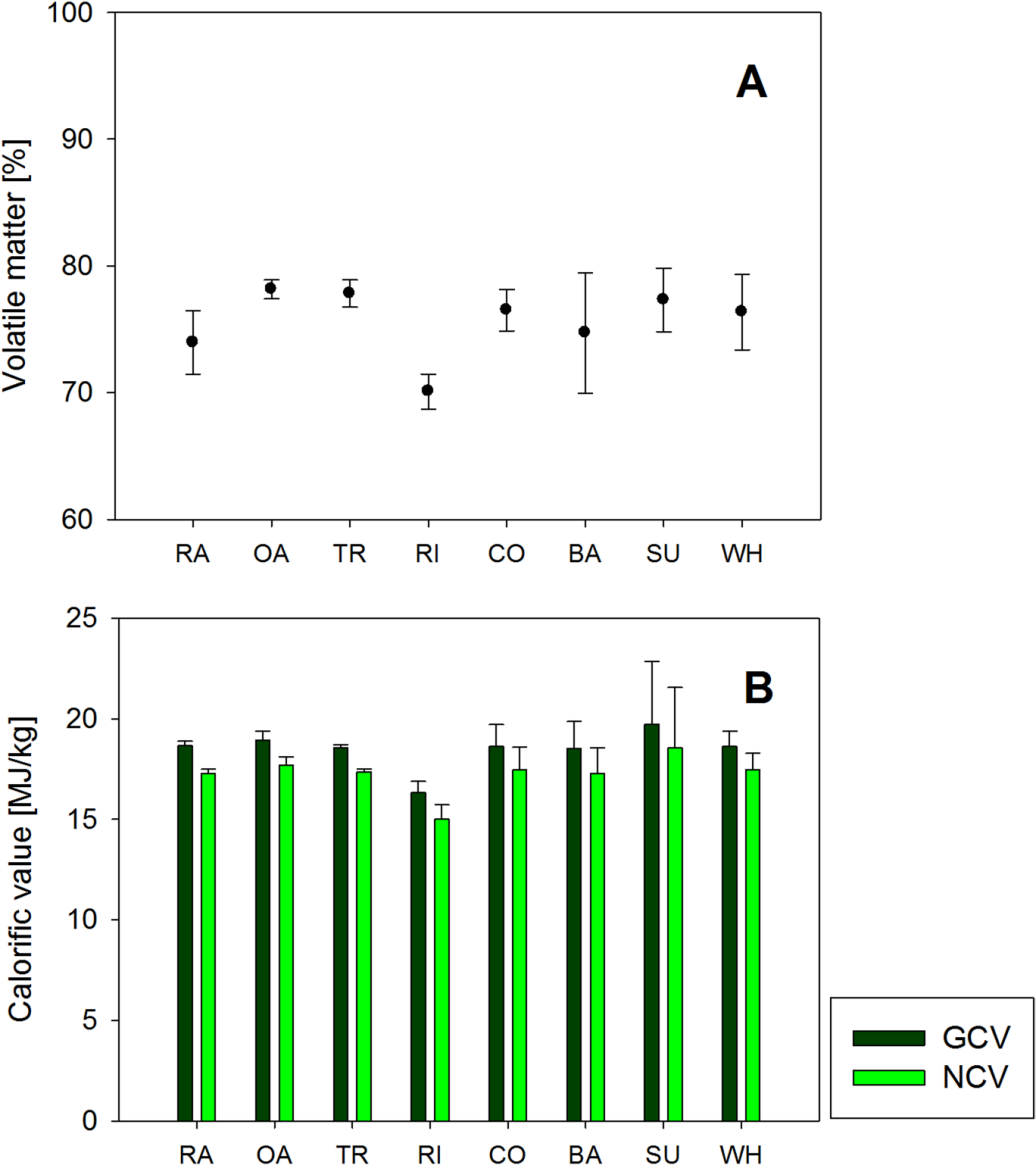
(**A**) Volatile matter in the studied biomass samples and (**B**) gross calorific value (GCV) and Net Calorific Value (NCV) of biomass assessed. Rapeseed (RA), oatmeal (OA), triticale (TR), rice (RI), corn (CO), barley (BA), sunflower (SU), and wheat (WH).

A high volatile matter content promotes combustion and improves the reactivity of biomass through secondary cracking and condensation reactions, whose products are also burned. However, due to the increased combustion rate, this can lead to incomplete combustion, generating carbon monoxide (CO) emissions instead of carbon dioxide (CO_2_), as well as other pollutants^41,42^. In the case of biomass boilers and stoves, volatile contents 75–85% are recommended^43–45^; for gasification processes, it should be 80–90%^46,47^, and in fluidized bed combustion systems, 65–75%^48–50^. Figure 4 shows that the recommended range of 75–80% for biomass boilers are OA straw, TR straw, CO straw, SU straw, and WH straw. In this regard, the use of RI straw would not be recommended, as it is 70%.

Regarding the calorific values (Fig. 3B), RI straw presents the lowest NCV, around 15.0 MJ kg^-1^, while SU straw undoubtedly shows the highest value, around 19 MJ kg^-1^. However, the remaining samples cannot be significantly distinguished from SU straw, as they may be like it or similar to BA straw, which has an NCV of around 17.0 MJ kg^-1^. Such values are higher than representative native shrubs of NW Spain and North-Central Portugal, with values ranging from 8.4 to 11.2 MJ kg^-1^^51^. Compared with other herbaceous agricultural residues reported in the literature, the gross calorific values (GCV) obtained for the straws analyzed in this study fall within the upper range typically reported for non-woody biomasses. Greenhouse crop residues, such as tomato, cucumber, and pepper plants, generally exhibit lower GCV values, typically ranging from 11.5 to 15.8 MJ kg^−1^^22^ mainly due to their higher ash and moisture contents. In contrast, the straws investigated here present higher GCV values, commonly between 16 and 20 MJ kg^−1^, which are comparable to those reported for other agricultural residues such as sunflower husks, shells, and mixed stem–floret fractions (≈17.5–20.2 MJ kg^−1^), as well as corn husk and stalk fractions (≈16.1–16.6 MJ kg^−1^). These results confirm that cereal and oilseed straws exhibit an energy performance closer to selected high-quality agricultural sub-fractions than to greenhouse residues, reinforcing their suitability as solid biofuels for thermochemical conversionRecently, banana peel waste was evaluated as possible bioenergy resource and value-added chemicals. Obtaining 15.6 MJ Kg^-1^ as NCV^10^, in the range of straw and greenhouses studied. The SU straw reached a higher heating value of 19.0 MJ kg, whereas the other types of straw analyzed ranged between 15.0 and 17.0 MJ kg^-1^. This positions SU straw at a level comparable to woody biomasses such as oak (19.8 MJ kg^-1^) or spruce (20.5 MJ kg^-1^) and even close to high-performing residues such as olive pits (22.0 MJ kg^-1^)^52–54^. In contrast, the other straws within the lower range reflect a more limited performance, falling below the typical values reported for many lignocellulosic biomasses in the literature (18.7–22.0 MJ kg^-1^). Such variability can be attributed to differences in elemental composition and ashes, since materials with a higher proportion of carbon and a lower inorganic fraction generally display greater energy density. In this context, while SU straw stands out as a competitive alternative compared to reference biomasses, the remaining straws present only moderate energy quality, which could be improved through pretreatment processes such as drying, densification, or blending with higher-calorific-value biomasses^55,56^.

### Elementary analysis (C, H, O, N, S, and Cl)

**Fig. 5:**
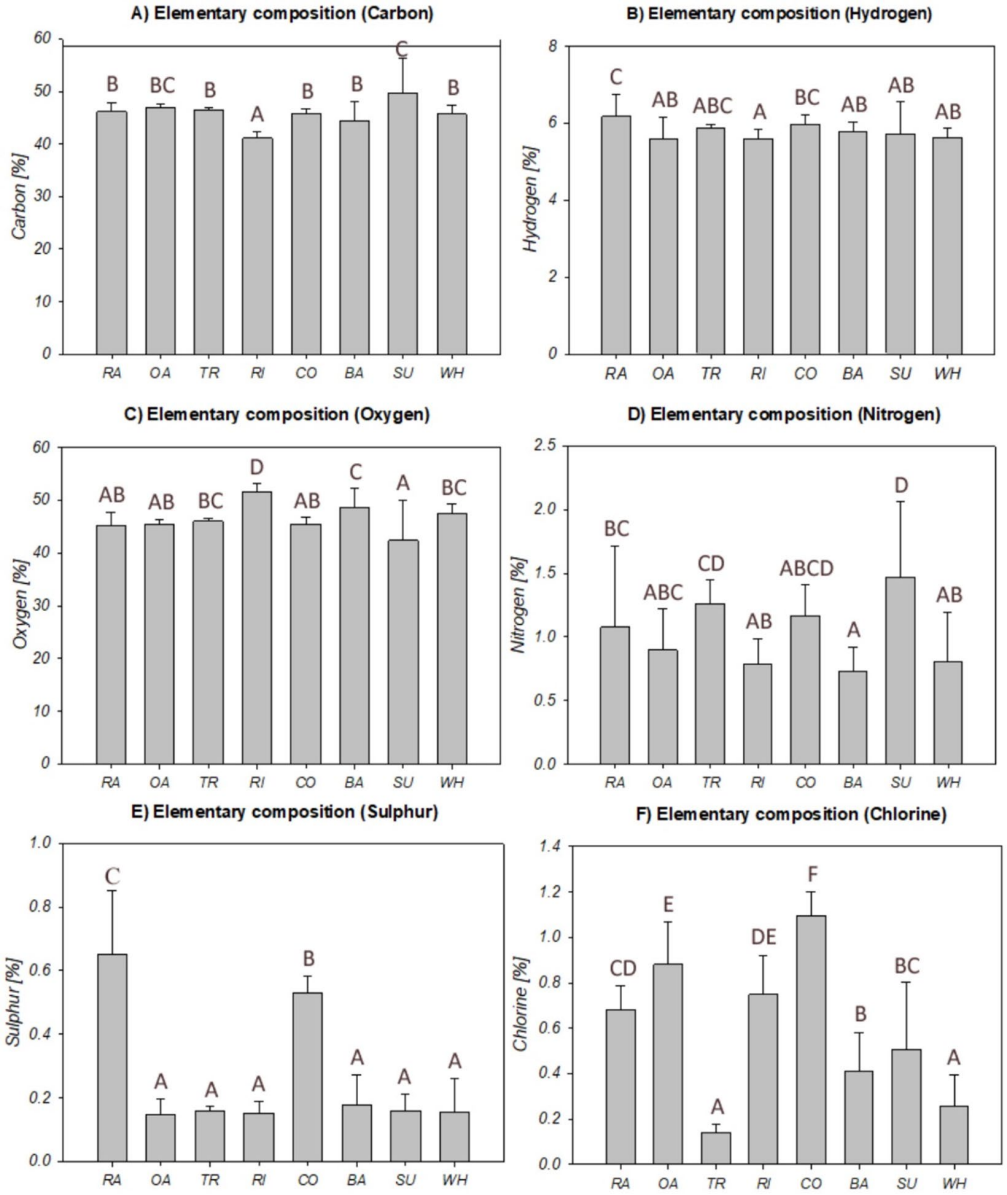
Elementary composition (%): (**A**) carbon, (**B**) hydrogen, (**C**) oxygen, (**D**) nitrogen, (**E**) sulfur, (**F**) chlorine. Rapeseed (RA), oatmeal (OA), triticale (TR), rice (RI), corn (CO), barley (BA), sunflower (SU), and wheat (WH).

The carbon content of the residues studied (Fig. 5A) ranges from approximately 40 to 50% d.b., with sunflower (SU) straw showing the highest values (≈50%) and rice (RI) straw the lowest (≈40%). These values are consistent with those reported for cereal and oilseed agricultural residues, as well as sunflower- and corn-derived materials, where carbon contents typically range between 42 and 52%, and confirm the strong link between higher carbon content and improved calorific performance observed in previous studies. The remaining samples fall within values close to the average. Carbon content is important in a biomass valorization study as a biofuel due to its close relationship with net calorific value. In the case of hydrogen (Fig. 5B), all the samples show similar values of 6%. The oxygen content (Fig. 5C) of the samples is 45%, with RI straw standing out as it has a higher value of 50%.

The nitrogen content (Fig. 5D) is important to consider, as it will determine the concentration of NOx generated during combustion, which must be considered as low as possible to minimize the impact of NOx emissions to comply with the European Regulation (2015/1189)^57^, Royal Decree (RD) 2017/1042^58^, and local normative. Nitrogen concentrations ranged from 0.7% to 1.5% d.b., with barley (BA) straw showing the lowest values and sunflower (SU) straw the highest. This range is comparable to that reported for other herbaceous agricultural residues and is generally higher than typical values for woody biomasses (< 0.5%), reflecting the higher protein content of straws and a potentially increased tendency for NOₓ formation during combustion, as also observed in previous studies on cereal and sunflower residues.

Similarly, sulphur concentration (Fig. 5E) determines the generation of SOx, which must also be considered as low as possible to minimize the impact of emissions to comply with the European Regulation (2015/1189)^59^, Royal Decree (RD) 2017/1042^58^, and local normative. Among the samples studied, most have values of 0.2%, with CO straw standing out at 0.5% and RA straw at 0.6%, which results showed slightly lower values than greenhouse residues [^19,37,60^]. Finally, chlorine content (Fig. 5F) is studied due to the formation of harmful compounds such as dioxins or furans, which are also regulated (RD 2013/815, and RD 2017/1042)^58^. In addition, chlorine can cause corrosion in boiler materials due to the formation of hydrochloric acid (HCl). Triticale and wheat straws present the lowest chlorine contents (≈0.2% d.b.), followed by barley (BA) straw (≈0.4%), whereas corn (CO) straw shows the highest concentration (≈1.1%), followed by oats (OA) straw (≈0.9%) and rice (RI) straw (≈0.8%). This wide variability is consistent with literature data for agricultural residues, where chlorine accumulation is strongly influenced by crop type, fertilization practices, and soil conditions, with corn- and oat-derived materials frequently exhibiting elevated chlorine levels compared to cereal straws.

The results obtained for the different straws analyzed (RA, OA, TR, RI, CO, BA, SU, and WH) indicate elemental compositions that generally fall within the ranges reported for woody biomasses and agricultural residues in the literature, albeit with some noteworthy differences. The carbon content of the samples (≈ 42–48%) is comparable to that observed in biomasses such as wood, bagasse, or nutshells (≈ 45–53%), while the proportions of hydrogen (≈ 5–7%) and oxygen (≈ 43–50%) also fall within similar intervals, though with greater variability attributable to the heterogeneity of agricultural residues compared with more homogeneous woody biomasses. The nitrogen content (≈ 0.5–2%) is generally higher than that reported for wood and bagasse (< 1%), which is consistent with the higher protein content characteristic of stems and straws and suggests an increased potential for NOx emissions during combustion. Finally, sulphur concentrations range between approximately 0.1 and 0.9% d.b., which is in line with values reported for non-woody agricultural biomasses and generally higher than those of woody fuels. The comparatively higher sulphur contents observed for rapeseed and corn straw are consistent with previous studies and can be attributed to crop-specific nutrient uptake and fertilization practices, with implications for SO₂ emissions during combustion, clearly above the values typically reported for woody biomasses, where they remain mostly at trace levels (< 0.1%), implying a higher potential for SO₂ formation^23,55,61,62^.

### Correlation study for calculating the calorific value

**Fig. 6:**
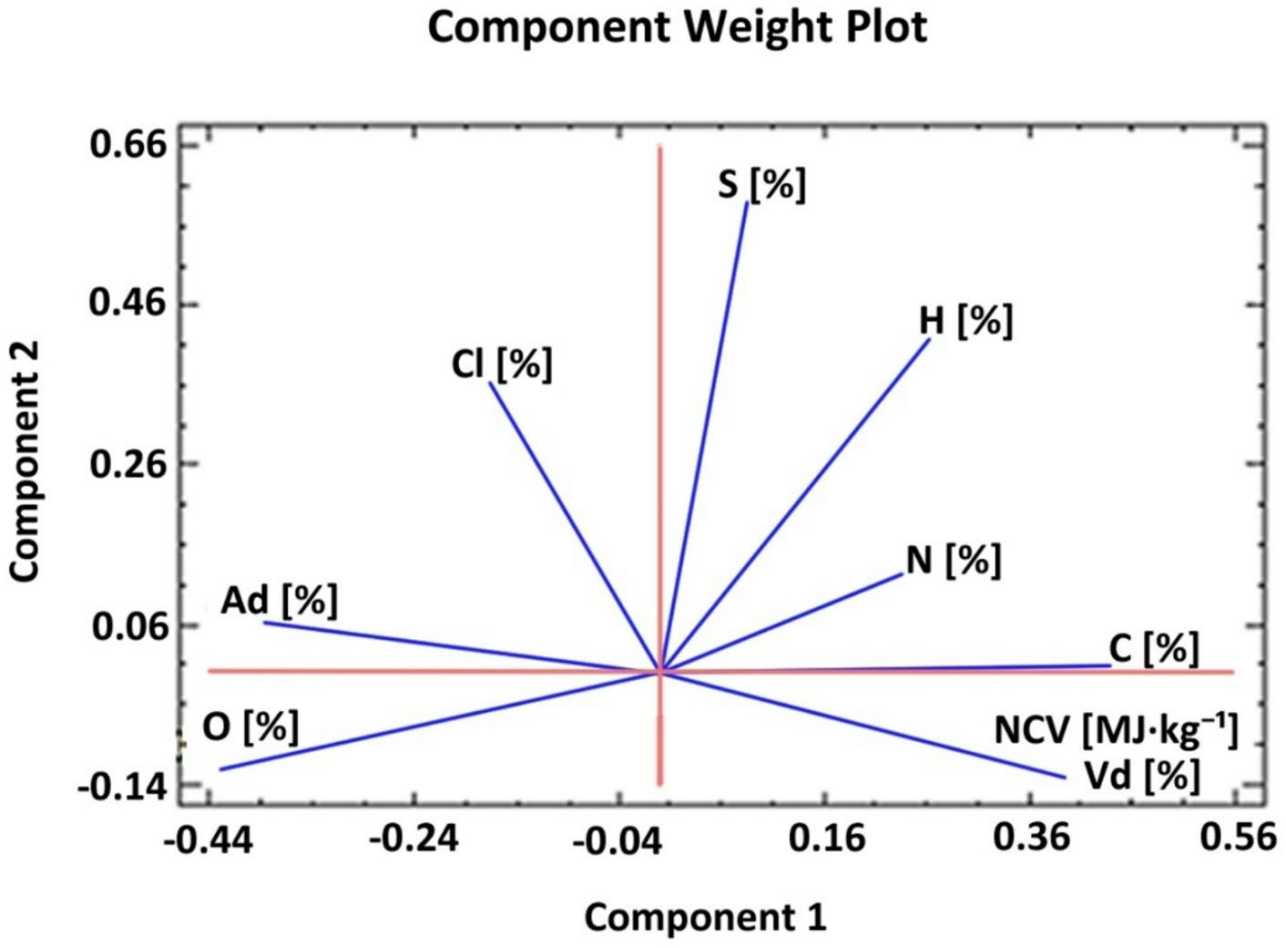
PCA of biomass combustion parameters: linking fuel properties to calorific value.

First, PCA was carried out to study how certain elements are related to the calorific capacity of the straw species studied (Fig. 6). This analysis considered the NCV, the ${V}_{d}$, ${A}_{d}$, and the C, H, O, N, Cl, and S. The analysis reveals a strong positive correlation between carbon content and volatile matter on a dry basis^13,19,63^. Carbon is the primary element responsible for the NCV, and the generated volatiles are closely related to the amount of carbon burned during combustion, as most of the volatiles released during biomass combustion are compounds derived from carbon. Numerous studies directly associate carbon content with calorific capacity, as well as with other structural components of biomass that serve as carbon sources, such as lignin and cellulose^3,13,19,61^. On the other hand, the ash content on a dry basis, oxygen, and to a lesser extent, the ambient temperature, show a negative correlation with the NCV of the biomass. This can be explained by the fact that ash content on a dry basis corresponds to the inorganic residue remaining after complete combustion of the biomass, composed of compounds such as calcium, potassium, and magnesium oxides. Oxygen may be associated with the formation of these oxides, but also with the biomass moisture, which has a detrimental effect on its calorific value^13,22,61^.

Based on the available data, empirical multivariate models were developed to estimate the net calorific value (NCV) from routinely measured compositional parameters. These models are intended as practical tools for rapid NCV screening, rather than as mechanistic representations of fuel chemistry. Due to the strong inter-correlation among biomass constituents (e.g. carbon, hydrogen, volatile matter, ash and heteroatoms), some regression coefficients may not strictly follow the expected physical contribution of individual elements when interpreted in isolation. In this context, variables such as chlorine should be regarded as proxy indicators linked to broader compositional patterns within the dataset, rather than as quality enhancing fuel components. Consequently, while the models show high predictive performance for the studied straws, their applicability outside similar biomass streams or regional conditions should be considered with caution.

**Fig. 7:**
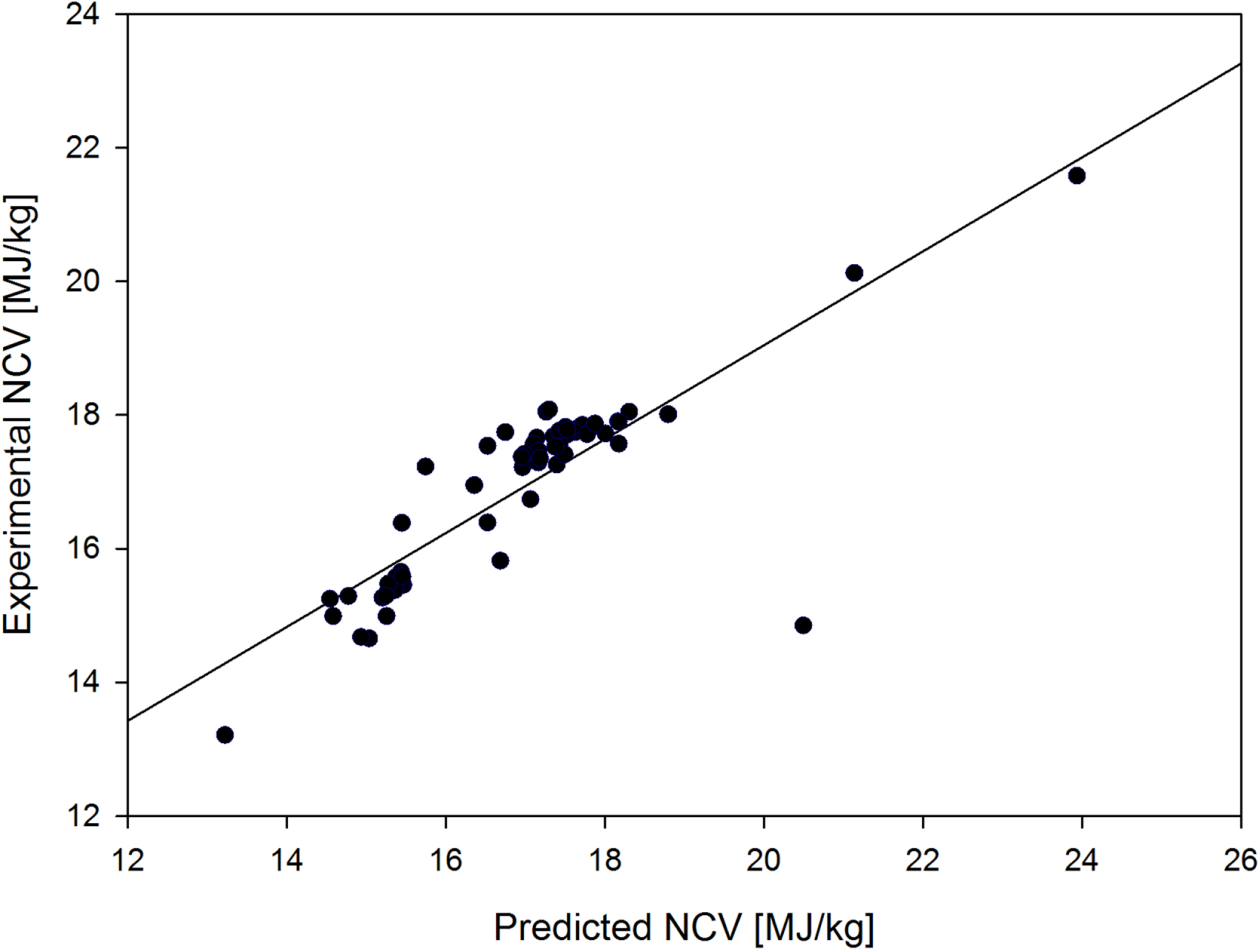
General model for straw’s net calorific value (NCV).

This enables the early elimination of samples that are unlikely to meet the desired criteria, thereby reducing research costs. Based on the results and previous models developed^13,61^, a general model (Table 1) was proposed to predict the NCV of the biomass derived from the straws analyzed in this work^3,13,19,61^. However, this model yields an R^2^ value of only 70% (Fig. 7). To improve predictive accuracy, individual models (Table 1) were also developed for each type of straw, achieving an R^2^ values exceeding 90%, except for RI straw (84%), BA straw (41%) and WE straw (57%).

**Table 1:** Models developed to predict the net calorific value (MJ kg^-1^).

Straw Type	Equation
General	$NCV (MJ kg^{-1})=-0.118A_{d}-0.220O+ 28.320$
	$${R}^{2}=0.7007$$
Sunflower	$NCV (MJ kg^{-1})=0.448C-3.734$
	$${R}^{2}=0.9676$$
Rapeseed	$NCV ( MJ kg^{-1})=0.316C-0.403H+5.071$
	$${R}^{2}=0.9997$$
Oats	$NCV (MJ kg^{-1})=-0.265{A}_{d}+0.335C+ 3.386$
	$${R}^{2}=0.9836$$
Triticale	$NCV (MJ kg^{-1})=0.146{V}_{d} + 2.530Cl+ 5.663$
	$${R}^{2}=0.9222$$
Rice	$NCV (MJ kg^{-1})=-0.131{A}_{d}+0.176C+8.546$
	$${R}^{2}=0.8461$$
Corn	$NCV (MJ kg^{-1})=- 0.302{A}_{d} + 4.360N+ 13.980$
	$${R}^{2}=0.9734$$
Barley	$NCV (MJ kg^{b-1})=-0.951{A}_{d}-0.417{V}_{d}+ 54.521$
	$${R}^{2}=0.4116$$
Wheat	$NCV (MJ kg^{-1})= 0.215O - 5.730S+ 8.146$
	$${R}^{2}=0.5694$$

**Fig. 8:**
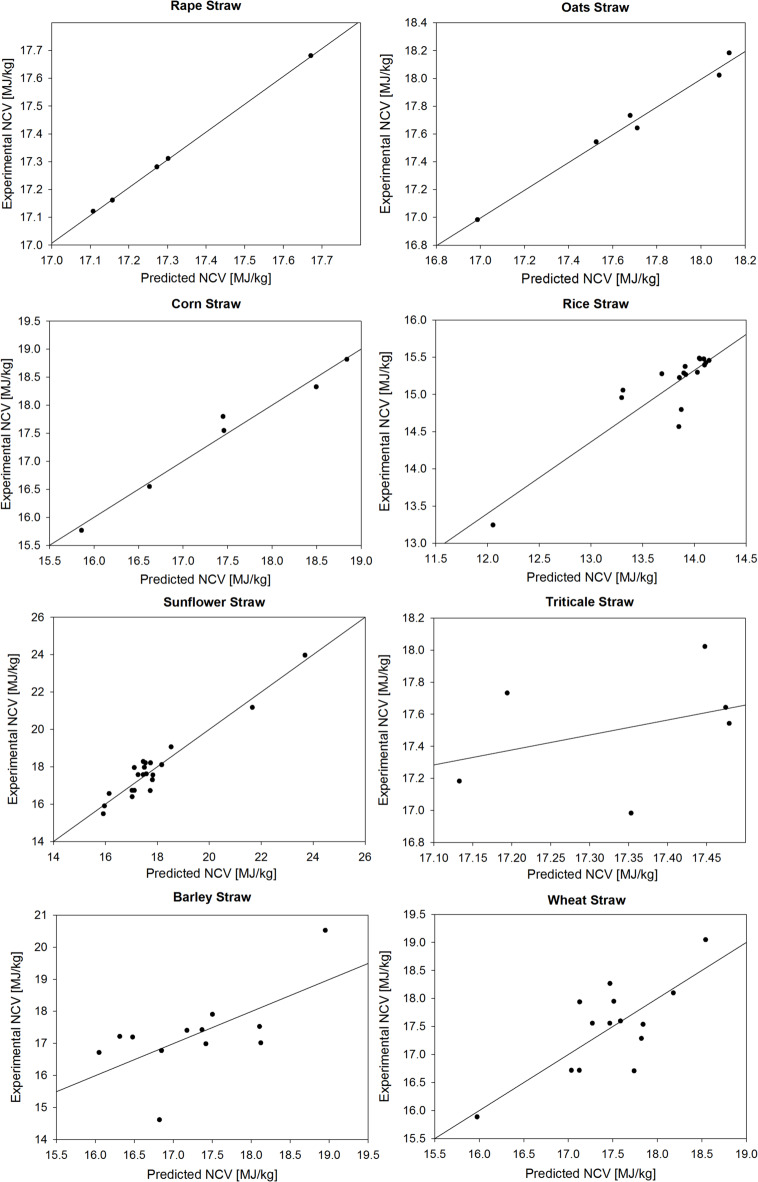
Prediction model for each straw species net calorific value (NCV). Rapeseed (RA), oatmeal (OA), triticale (TR), rice (RI), corn (CO), barley (BA), sunflower (SU) and wheat (WH).

For most straw species, the predictive models developed exhibit high coefficients of determination (R^2^ > 0.90), confirming a strong linear relationship between NCV and the selected compositional variables (Fig. 8). Sunflower (SU) straw is the only case in which an R^2^ above 90% (97%) was achieved using a single predictor, namely carbon content (C), highlighting its relatively homogeneous composition. In contrast, the models developed for barley (BA) and wheat (WH) straw show lower R^2^ values (0.41 and 0.57, respectively). This reduced performance is attributed to the higher physicochemical heterogeneity of these residues, which may arise from differences in harvesting practices, soil contamination, and field-specific growing conditions among samples. Such variability weakens simple linear correlations with NCV and limits the predictive capability of reduced-variable models. Consequently, while NCV can be reliably estimated for most straws using the proposed correlations, the models for BA and WH should be interpreted with caution, and their accuracy could be improved by incorporating additional explanatory variables or more homogeneous sample subsets. The equations for the general model and the species-specific models are summarized in Table 1.

The Table 2 presents the Mean Bias Error (MBE) and Mean Absolute Error (MAE) of the model predicting the NCV (MJ kg^-1^ ) for the selected biomass types. Overall, the general model exhibits relatively low error values (MBE = 0.0254 MJ/kg, MAE = 0.4642 MJ kg^-1^), indicating reasonable predictive accuracy across all biomass categories. Among individual biomasses, RI shows the largest deviations (MBE = 1.3331 MJ kg^-1^, MAE = 1.3331 MJ kg^-1^), suggesting that the model tends to overestimate its NCV. Other biomasses, such as SU, RA, OA, CO, and WH, display minor errors, with MAE values generally below 0.5 MJ kg^-1^, reflecting reliable predictions. BA shows a moderate MAE (0.7427 MJ kg^-1^) and slight negative bias (MBE = -0.0085 MJ kg^-1^), indicating small underestimation. Notably, the general model’s error metrics are comparable to most individual biomasses, supporting its potential applicability for estimating NCV across diverse feedstocks.

**Table 2:** Mean bias error (MBE) and mean absolute error (MAE).

	MBE (MJ kg^-1^)	MAE (MJ kg^-1^)
General	0.0254	0.4642
Sunflower	0.0017	0.4831
Rapeseed	0.0057	0.0057
Oats	− 0.0058	0.0431
Triticale	0.1674	0.2920
Rice	1.3331	1.3331
Corn	0.0012	0.1362
Barley	− 0.0085	0.7427
Wheat	0.0003	0.4076

### Comparing the characterization of the straws with the biofuel standard

To promote the use of straw residues in thermochemical applications, it is important not only to characterize them as solid biofuels but also to classify them within existing quality standards. In this regard, the European Committee for Standardization (CEN) has developed standards for solid biofuel biomass in the form of the technical standardization committee (CEN/TC 335 Solid Biofuels), which classifies them according to their energy parameters. This allows biomass to be classified as higher or lower quality for use in bioenergy applications. The purpose of this series of standards is to provide unambiguous classification guidelines for solid biofuels. These guidelines serve to enhance trade, improve understanding between sellers and buyers, and facilitate communication with equipment manufacturers^64^. These product standards classify solid biofuels used in commercial buildings, such as residential and small commercial buildings, as well as public and industrial power generation applications. These applications require fuels with specified qualities, expressed as quality classes such as A or B CTN 164 Solid Biofuels^64^. Specifically, to compare straw residues with the chemical and energy parameters indicated in the quality standards, parameters relating to pellets and/or briquettes and the physical qualities derived from their manufacture and production have been avoided. Thus, this discussion is intended to serve as a guide to their potential as a preliminary step to their proper treatment and transformation into solid biofuels, primarily pellets and briquettes. In this framework and according to the CTN 164 Solid Biofuels Standard for non-wood biomass pellets and briquettes^65,66^, straw residues collected in the field should be subjected to a drying pre-treatment to reduce their moisture content below 12 and 15%.

In terms of ash content, the average results (Table 3) for the OA, TR, and RI samples analyzed were within class A, whereas the RA, BA, SU, and WH samples were consistent with class B. However, the ash content of RI straw was very high (15.28%), placing it outside the proposed classification (≤ 10%). The high ash content of this type of straw can negatively affect energy yield and combustion behavior, generating deposits and slagging risk. In such cases, blending RI straw biomass with other low-ash species is an effective solution for producing higher-quality solid biofuels. Additionally, efficient combustion equipment must be used to remove ash from combustion gases, as is the case with coal combustion^67^, to eliminate or reduce particle pollution.

**Table 3:** Comparison of the average results for the straw to the specification for the pellet and briquettes produced from non-woody biomass, CTN 164 solid biofuels^65^ and CTN 164 solid biofuels^66^.

Parameter	Unit	Specifications		Straws characterized in this study							
		A	B	RA	OA	TR	RI	CO	BA	SU	WH
M_ad_	% (w/w) wet basis	< 12	< 15								
A_d_	% (w/w) dry basis	< 6.0	< 10	6.15 ± 1.42	5.43 ± 1.21	5.03 ± 0.74	15.28 ± 2.16	5.30 ± 1.26	6.41 ± 2.06	7.4 ± 3.25	6.10 ± 1.84
NCV	MJ kg^-1^ as received	> 14.5	> 14.5	17.27 ± 0.22	17.68 ± 0.47	17.35 ± 0.15	15.00 ± 0.56	17.46 ± 1.09	17.26 ± 1.34	18.55 ± 3.13	17.48 ± 0.77
N	% (w/w) dry basis	< 1.5	< 2.0	1.08 ± 1.08	0.90 ± 0.32	1.26 ± 0.19	0.79 ± 0.20	1.17 ± 0.25	0.73 ± 0.19	1.47 ± 0.59	0.81 ± 0.39
S	% (w/w) dry basis	< 0.20	< 0.30	0.65 ± 0.20	0.15 ± 0.05	0.16 ± 0.02	0.15 ± 0.04	0.53 ± 0.05	0.18 ± 0.10	0.16 ± 0.05	0.15 ± 0.11
Cl	% (w/w) dry basis	< 0.10	< 0.30	0.68 ± 0.10	0.88 ± 0.18	0.14 ± 0.04	0.75 ± 0.17	1.10 ± 0.10	0.41 ± 0.17	0.51 ± 0.30	0.26 ± 0.14
As	mg/kg dry basis	< 1	< 1				0.35 ± 0.29				
Cd		< 0.5	< 0.5				0.03 ± 0.03				
Cr		< 50	< 50				3.19 ± 3.65				
Cu		< 20	< 20				0.66 ± 0.79				
Pb		< 10	< 10				0.43 ± 0.64				
Zn		< 100	< 100				3.03 ± 3.46				
Ash fusibility	°C	Should be declared	Should be declared	1310 ± 18	1146 ± 68	1363 ± 27	1288 ± 34	1148 ± 42	1157 ± 66	1411 ± 66	1181 ± 31

In the context of energy use, it is interesting to note that all eight types of straw analyzed (Table 3) showed an NCV above the established standard of 14.5 MJ kg^-1^ (limit indicated for classes A and B). This energetic characteristic provides them with a high potential for energy valorization, making them suitable for direct use and for conversion into densified solid biofuels such as pellets and briquettes. Similarly, it is noteworthy that all samples had nitrogen values below the maximum established in category A (1.5% w/w). The same can be said for sulphur content, except for RA and CO straw, whose values exceed 0.3% w/w for class B (Table 3). However, more heterogeneous results were found in the chlorine analyses. Thus, in terms of chlorine content alone, TR is very close to class A, while WH would correspond to class B, and the rest of the straws would be above the established limit (0.3% w/w). According to the CTN 164 Solid Biofuels Standard^65,66^, no value limits for the melting temperature have been established, although these temperatures must be declared. The results for all the samples analyzed showed high ash melting temperatures of 1100 °C or above.

Among the parameters of major interest for solid biofuel producers are calorific value, moisture and ash content. Furthermore, minor elements also have maximum limits according to regulatory standards^65,66^, as in some cases they may be a cause of environmental concern. In this study, this analysis was not carried out on the samples analyzed, as they are biomasses of agricultural origin and have not been chemically treated, so the minor elements should be found in values lower than those established by the standard^64^.

## Conclusions

This study presents a harmonized and statistically robust characterization of eight agricultural straw residues from Southern Spain based on the analysis of 75 independent samples, evaluating their suitability as solid biofuels for thermochemical applications. The use of fully standardized CEN/ISO methodologies across all samples enables direct comparability among different straw types and reduces methodological bias, addressing a relevant gap in existing literature that often relies on heterogeneous datasets. In particular, this work provides consolidated and original physicochemical and energetic data for rapeseed and triticale straws, which remain poorly represented in previous bioenergy studies despite their increasing agricultural relevance. Ash content exhibited significant variability among species, with oat, triticale and corn straw (5.03–5.43%) meeting the requirements for Class A fuels under EN ISO 17225, while rapeseed, barley, sunflower and wheat straw (6.10–7.44%) were classified as Class B. All straws showed net calorific values (NCV) ranging from 15 to 19 MJ kg^−1^, determined under standardized analytical conditions according to ISO 18125:2017 at a moisture content not exceeding 20%, confirming their high potential for energy valorization and their suitability for direct combustion or densification into pellets and briquettes. Nitrogen contents remained below 1.5% w/w for all samples, fulfilling Class A specifications, while sulphur and chlorine contents highlighted the need for appropriate fuel management strategies, such as blending or pretreatment, for certain straw types. In addition, NCV-oriented empirical linear correlations were developed based on proximate and ultimate analyses. Species-specific models achieved excellent predictive performance (R^2^ > 0.90) for most straws, demonstrating their potential as practical tools for rapid energy-quality screening using routinely measured parameters. Lower correlations observed for barley and wheat straws reflect their higher physicochemical heterogeneity and underline the importance of biomass uniformity when applying simplified predictive models.

The limitations of this study should also be acknowledged. Although the dataset is extensive, it is geographically restricted to Andalusia (Southern Spain) and to a single harvesting year, which may limit the direct extrapolation of the results to other regions or agronomic conditions. The NCV prediction models are empirical and linear, and their performance may decrease when applied to biomass produced under different soil, climate, or management practices. Moreover, detailed mineralogical characterization of ash-forming elements was beyond the scope of this work, constraining the mechanistic interpretation of ash fusibility behavior. Future research should therefore focus on extending the dataset across regions and years, incorporating ash chemistry, and validating the proposed models at pilot and industrial scale. Overall, the results confirm that agricultural straw residues represent a promising renewable feedstock for solid biofuels in Mediterranean regions, while providing practical data and tools that support their integration into regional bioenergy systems.

## Supplementary Information

Below is the link to the electronic supplementary material.

Supplementary Material 1

## Data Availability

We have incorpored all reserch data in the original and supplementary manuscript.
