# Supplementary material for: Bioenergy potential of agricultural straw residues from Southern Spain
Source: Sci Rep. 2026 Apr 21;16:18655. doi: 10.1038/s41598-026-46840-z (PMC13269490; doi:10.1038/s41598-026-46840-z)
Supplement: Supplementary file 1 — Supplementary Material 1 [file 41598_2026_46840_MOESM1_ESM.docx]

#

# Supplementary material

**Bioenergy Potential of Agricultural Straw Residues from Southern Spain**

M. Guadalupe Pinna-Hernández ^a,b,*^, Rubén López Pastor ^b^, Manuel J. Díaz Villanueva ^c^, José Luis Casas López ^a,b^, Francisco Gabriel Acien Fernández ^a,b^

^a^ *Department of Chemical Engineering, University of Almería, Carretera de Sacramento s/n 04120 La Canada de San Urbano, Almería, Spain*

^b^ *Solar Energy Research Centre (CIESOL), Joint Centre University of Almería-CIEMAT, Almería, 04120, Spain*

^c^ *Department of Chemical Engineering and Food Technology, Wine and Agrifood Research Institute (IVAGRO), University of Cádiz - International Campus of Excellence (ceiA3), 11510 Puerto Real, Cádiz, Spain.*

*Corresponding author

*E-mail address:* gpinnahernandez@ual.es (M. Guadalupe Pinna Hernández).

This document provides more detailed information on the paper mentioned above.

The following information is included:

| **Content** |  | **Page** |
| --- | --- | --- |
| Table S1 | Cultivated area (in hectares) and production (in tons) of straw crops in 2023 [11] | 3 |
| Table S2 | Location of origin and number of straw samples | 4 |
| Table S3 | Statical description of the proximate parameters of the straw crop residues | 5 |
| Table S4 | Statical description of elementary analysis | 6-7 |
| Table S5 | Statical description of ash fusibility | 8 |
| Table S6 | ANOVA results of the proximate parameters of the straw crop residues | 9 |
| Table S7 | ANOVA results of elementary analysis | 10 |
| Table S8 | ANOVA results of ash fusibility | 11 |
| Table S9 | Moisture Content | 12 |
| Figure S1 | Example screenshot of the pyramidal ash pellet at different characteristic temperatures: (1) at DT temperature, (2) at ST, (3) at HT, and (4) at FT | 13 |

**Table S1.** Cultivated area (in hectares) and production (in tons) of straw crops in 2023 [11]

| **Area** | **Item** | **Element** | **Value** | **Unit** |
| --- | --- | --- | --- | --- |
| Spain | Barley | Area harvested | 2342260 | ha |
|  | Barley | Production | 3757570 | tons |
|  | Cereals | Area harvested | 5610 | ha |
|  | Cereals | Production | 13400 | tons |
|  | Maize (corn) | Area harvested | 239900 | ha |
|  | Maize (corn) | Production | 2835360 | tons |
|  | Mixed grain | Area harvested | 37130 | ha |
|  | Mixed grain | Production | 32940 | tons |
|  | Oatmeal | Area harvested | 466760 | ha |
|  | Oatsmeal | Production | 464140 | tons |
|  | Rapeseed | Area harvested | 111860 | ha |
|  | Rapeseed | Production | 170840 | tons |
|  | Rice | Area harvested | 55120 | ha |
|  | Rice | Production | 334100 | tons |
|  | Sunflower | Area harvested | 781710 | ha |
|  | Sunflower | Production | 849400 | tons |
|  | Triticale | Area harvested | 249430 | ha |
|  | Triticale | Production | 288190 | tons |
|  | Wheat | Area harvested | 1957760 | ha |
|  | Wheat | Production | 4049230 | tons |
|  |  |  |  |  |

**Table S2.** Location of origin and number of straw samples

| **Straw** | **Location** | **Samples** |
| --- | --- | --- |
| Rapeseed straw (RA) | Jaén | 6 |
| Oats straw (OA) | Sevilla | 6 |
| Triticale straw (TR) | Córdoba | 6 |
| Rice straw (RI) | Sevilla | 18 |
| Corn straw (CO) | Sevilla | 6 |
| Barley straw (BA) | Jaén | 12 |
| Sunflower straw (SU) | Córdoba | 7 |
| Wheat straw (WH) | Sevilla | 14 |

**Table S3.** Statical description of the proximate parameters of the straw crop residues. Rapeseed (RA), oats (OA), triticale (TR), rice (RI), corn (CO), barley (BA), sunflower (SU), and wheat (WH).

|  | **A_d (%)** | | | | | | | | |
| --- | --- | --- | --- | --- | --- | --- | --- | --- | --- |
|  | **RA** | **OA** | **TR** | **RI** | **CO** | **BA** | **SU** | **WH** | **TOT** |
| Average | 6.15 | 5.43 | 5.03 | 15.28 | 5.30 | 6.41 | 7.44 | 6.10 | 7.14 |
| Median | 5.78 | 5.18 | 4.99 | 14.59 | 5.33 | 5.91 | 8.50 | 5.41 | 5.59 |
| Standard desviation | 1.42 | 1.21 | 0.74 | 2.16 | 1.26 | 2.06 | 3.25 | 1.84 | 0.77 |
| Variance | 2.02 | 1.47 | 0.55 | 4.66 | 1.58 | 4.23 | 10.54 | 3.39 | 10.02 |
| Max. Value | 8.69 | 7.50 | 5.99 | 22.50 | 6.90 | 9.95 | 10.90 | 9.32 | 22.50 |
| Min. Value | 4.72 | 3.95 | 3.98 | 13.60 | 3.19 | 3.85 | 2.98 | 3.80 | 2.98 |
|  | **V_d (%)** | | | | | | | | |
|  | **RA** | **OA** | **TR** | **RI** | **CO** | **BA** | **SU** | **WH** | **TOT** |
| Average | 73.96 | 78.15 | 77.82 | 70.09 | 76.51 | 74.71 | 77.30 | 76.36 | 75.61 |
| Median | 74.49 | 77.99 | 78.00 | 70.44 | 76.55 | 76.00 | 77.82 | 76.11 | 76.33 |
| Standard desviation | 2.51 | 0.74 | 1.06 | 1.38 | 1.63 | 4.77 | 2.49 | 2.97 | 1.30 |
| Variance | 6.30 | 0.54 | 1.13 | 1.90 | 2.67 | 22.71 | 6.21 | 8.85 | 52.62 |
| Max. Value | 76.45 | 79.58 | 78.90 | 72.13 | 78.53 | 79.98 | 80.15 | 80.42 | 80.42 |
| Min. Value | 70.80 | 77.50 | 76.00 | 67.70 | 73.84 | 64.58 | 72.81 | 70.71 | 64.58 |
|  | **GCV (MJ/kg)** | | | | | | | | |
|  | **RA** | **OA** | **TR** | **RI** | **CO** | **BA** | **SU** | **WH** | **TOT** |
| Average | 18.67 | 18.92 | 18.57 | 16.33 | 18.62 | 18.53 | 19.73 | 18.63 | 18.50 |
| Median | 18.69 | 18.99 | 18.62 | 16.53 | 18.66 | 18.45 | 19.06 | 18.79 | 18.68 |
| Standard desviation | 0.22 | 0.47 | 0.15 | 0.56 | 1.09 | 1.34 | 3.13 | 0.77 | 0.96 |
| Variance | 0.05 | 0.22 | 0.02 | 0.32 | 1.20 | 1.78 | 9.79 | 0.59 | 10.95 |
| Max. Value | 18.95 | 19.44 | 18.75 | 16.73 | 19.99 | 21.76 | 25.50 | 19.65 | 25.50 |
| Min. Value | 18.35 | 18.07 | 18.38 | 14.34 | 16.93 | 15.88 | 16.74 | 17.12 | 14.34 |
|  | **NCV (MJ/kg)** | | | | | | | | |
|  | **RA** | **OA** | **TR** | **RI** | **CO** | **BA** | **SU** | **WH** | **TOT** |
| Average | 17.27 | 17.68 | 17.35 | 15.00 | 17.46 | 17.26 | 18.55 | 17.48 | 17.26 |
| Median | 17.22 | 17.69 | 17.40 | 15.28 | 17.67 | 17.19 | 18.19 | 17.55 | 17.48 |
| Standard desviation | 0.22 | 0.42 | 0.16 | 0.73 | 1.13 | 1.31 | 3.01 | 0.80 | 0.92 |
| Variance | 0.05 | 0.17 | 0.02 | 0.53 | 1.28 | 1.72 | 9.08 | 0.63 | 9.29 |
| Max. Value | 17.68 | 18.18 | 17.52 | 15.48 | 18.81 | 20.52 | 23.95 | 19.04 | 23.95 |
| Min. Value | 17.08 | 16.98 | 17.13 | 13.02 | 15.76 | 14.60 | 15.46 | 15.88 | 13.02 |

**Table S4.** Statical description of elementary analysis. Rapeseed (RA), oats (OA), triticale (TR), rice (RI), corn (CO), barley (BA), sunflower (SU), and wheat (WH).

|  | **C_d (%)** | | | | | | | | |
| --- | --- | --- | --- | --- | --- | --- | --- | --- | --- |
|  | **RA** | **OA** | **TR** | **RI** | **CO** | **BA** | **SU** | **WH** | **TOT** |
| Average | 46.14 | 46.99 | 46.53 | 41.10 | 45.81 | 44.39 | 49.73 | 45.70 | 45.80 |
| Median | 46.69 | 46.96 | 46.44 | 41.41 | 45.55 | 45.98 | 47.51 | 46.06 | 46.25 |
| Standard desviation | 1.76 | 0.68 | 0.44 | 1.24 | 0.90 | 3.64 | 6.62 | 1.71 | 2.07 |
| Variance | 3.11 | 0.47 | 0.20 | 1.53 | 0.80 | 13.26 | 43.78 | 2.92 | 224.06 |
| Max. Value | 47.38 | 47.95 | 47.22 | 42.33 | 46.91 | 47.10 | 61.23 | 48.05 | 61.23 |
| Min. Value | 42.76 | 46.01 | 46.10 | 36.70 | 44.94 | 36.80 | 43.90 | 41.75 | 36.70 |
|  | **H_d (%)** | | | | | | | | |
|  | **RA** | **OA** | **TR** | **RI** | **CO** | **BA** | **SU** | **WH** | **TOT** |
| Average | 6.18 | 5.61 | 5.86 | 5.60 | 5.98 | 5.78 | 5.73 | 5.63 | 5.80 |
| Median | 6.19 | 5.76 | 5.87 | 5.66 | 6.04 | 5.82 | 5.69 | 5.69 | 5.79 |
| Standard desviation | 0.57 | 0.54 | 0.12 | 0.26 | 0.23 | 0.24 | 0.82 | 0.24 | 0.24 |
| Variance | 0.32 | 0.29 | 0.01 | 0.07 | 0.05 | 0.06 | 0.68 | 0.06 | 0.05 |
| Max. Value | 6.80 | 6.15 | 6.05 | 5.86 | 6.19 | 6.11 | 7.41 | 5.95 | 7.41 |
| Min. Value | 5.31 | 4.92 | 5.73 | 4.94 | 5.57 | 5.40 | 4.95 | 5.08 | 4.92 |
|  | **N_d (%)** | | | | | | | | |
|  | **RA** | **OA** | **TR** | **RI** | **CO** | **BA** | **SU** | **WH** | **TOT** |
| Average | 1.08 | 0.90 | 1.26 | 0.79 | 1.17 | 0.73 | 1.47 | 0.81 | 1.02 |
| Median | 0.90 | 0.77 | 1.21 | 0.81 | 1.12 | 0.74 | 1.40 | 0.65 | 0.85 |
| Standard desviation | 0.64 | 0.32 | 0.19 | 0.20 | 0.25 | 0.19 | 0.59 | 0.39 | 0.18 |
| Variance | 0.41 | 0.10 | 0.04 | 0.04 | 0.06 | 0.03 | 0.35 | 0.15 | 0.02 |
| Max. Value | 2.23 | 1.45 | 1.51 | 1.05 | 1.51 | 1.10 | 2.29 | 1.68 | 2.29 |
| Min. Value | 0.56 | 0.59 | 1.05 | 0.09 | 0.91 | 0.45 | 0.51 | 0.49 | 0.09 |
|  | **S_d (%)** | | | | | | | | |
|  | **RA** | **OA** | **TR** | **RI** | **CO** | **BA** | **SU** | **WH** | **TOT** |
| Average | 0.65 | 0.15 | 0.16 | 0.15 | 0.53 | 0.18 | 0.16 | 0.15 | 0.27 |
| Median | 0.70 | 0.14 | 0.16 | 0.14 | 0.52 | 0.15 | 0.15 | 0.12 | 0.15 |
| Standard desviation | 0.20 | 0.05 | 0.02 | 0.04 | 0.05 | 0.10 | 0.05 | 0.11 | 0.06 |
| Variance | 0.04 | 0.00 | 0.00 | 0.00 | 0.00 | 0.01 | 0.00 | 0.01 | 0.00 |
| Max. Value | 0.86 | 0.21 | 0.18 | 0.29 | 0.62 | 0.31 | 0.24 | 0.50 | 0.86 |
| Min. Value | 0.32 | 0.10 | 0.13 | 0.12 | 0.48 | 0.03 | 0.09 | 0.08 | 0.03 |
|  | **O_d (%)** | | | | | | | | |
|  | **RA** | **OA** | **TR** | **RI** | **CO** | **BA** | **SU** | **WH** | **TOT** |
| Average | 17.27 | 17.68 | 17.35 | 15.00 | 17.46 | 17.26 | 18.55 | 17.48 | 17.26 |
| Median | 17.22 | 17.69 | 17.41 | 15.28 | 17.67 | 17.19 | 18.19 | 17.55 | 17.48 |
| Standard desviation | 0.22 | 0.42 | 0.16 | 0.73 | 1.13 | 1.31 | 3.01 | 0.80 | 0.92 |
| Variance | 0.05 | 0.18 | 0.02 | 0.53 | 1.28 | 1.71 | 9.09 | 0.63 | 9.30 |
| Max. Value | 17.08 | 16.98 | 17.13 | 13.02 | 15.76 | 14.60 | 15.46 | 15.88 | 17.13 |
| Min. Value | 17.68 | 18.18 | 17.52 | 15.48 | 18.81 | 20.51 | 23.95 | 19.04 | 15.48 |
|  | **Cl_d (%)** | | | | | | | | |
|  | **RA** | **OA** | **TR** | **RI** | **CO** | **BA** | **SU** | **WH** | **TOT** |
| Average | 0.68 | 0.88 | 0.14 | 0.75 | 1.10 | 0.41 | 0.51 | 0.26 | 0.59 |
| Median | 0.68 | 0.88 | 0.15 | 0.75 | 1.08 | 0.38 | 0.54 | 0.23 | 0.61 |
| Standard desviation | 0.10 | 0.18 | 0.04 | 0.17 | 0.10 | 0.17 | 0.30 | 0.14 | 0.08 |
| Variance | 0.01 | 0.03 | 0.00 | 0.03 | 0.01 | 0.03 | 0.09 | 0.02 | 0.00 |
| Max. Value | 0.84 | 1.20 | 0.18 | 0.98 | 1.27 | 0.91 | 0.82 | 0.60 | 1.27 |
| Min. Value | 0.56 | 0.64 | 0.09 | 0.33 | 0.98 | 0.26 | 0.09 | 0.10 | 0.09 |

**Table S5.** Statical description of ash fusibility. Rapeseed (RA), oats (OA), triticale (TR), rice (RI), corn (CO), barley (BA), sunflower (SU), and wheat (WH).

|  | **DT (ºC)** | | | | | | | | |
| --- | --- | --- | --- | --- | --- | --- | --- | --- | --- |
|  | **RA** | **OA** | **TR** | **RI** | **CO** | **BA** | **SU** | **WH** | **TOT** |
| Average | 1013 | 1030 | 1103 | 1228 | 1002 | 964 | 980 | 975 | 1037 |
| Median | 997 | 1027 | 1080 | 1206 | 995 | 951 | 992 | 972 | 996 |
| Standard desviation | 52 | 80 | 68 | 46 | 69 | 114 | 65 | 27 | 26 |
| Variance | 2662 | 6413 | 4676 | 2151 | 4796 | 13064 | 4225 | 740 | 14183960 |
| Max. Value | 946 | 909 | 1036 | 1161 | 897 | 798 | 898 | 920 | 1161 |
| Min. Value | 1085 | 1120 | 1186 | 1301 | 1095 | 1150 | 1085 | 1018 | 1018 |
|  | **ST (ºC)** | | | | | | | | |
|  | **RA** | **OA** | **TR** | **RI** | **CO** | **BA** | **SU** | **WH** | **TOT** |
| Average | 1157 | 1105 | 1237 | 1266 | 1087 | 1089 | 1084 | 1047 | 1134 |
| Median | 1159 | 1090 | 1239 | 1270 | 1098 | 1116 | 1110 | 1044 | 1113 |
| Standard desviation | 33 | 66 | 24 | 36 | 37 | 84 | 66 | 32 | 22 |
| Variance | 1084 | 4341 | 585 | 1291 | 1344 | 7097 | 4417 | 1055 | 5510772 |
| Max. Value | 1103 | 1032 | 1200 | 1182 | 1029 | 922 | 1005 | 985 | 1200 |
| Min. Value | 1197 | 1190 | 1273 | 1318 | 1129 | 1195 | 1175 | 1095 | 1095 |
|  | **HT (ºC)** | | | | | | | | |
|  | **RA** | **OA** | **TR** | **RI** | **CO** | **BA** | **SU** | **WH** | **TOT** |
| Average | 1310 | 1146 | 1363 | 1288 | 1148 | 1157 | 1411 | 1181 | 1250 |
| Median | 1308 | 1153 | 1373 | 1291 | 1162 | 1180 | 1450 | 1188 | 1239 |
| Standard desviation | 18 | 68 | 27 | 34 | 42 | 66 | 66 | 31 | 20 |
| Variance | 314 | 4632 | 733 | 1147 | 1793 | 4401 | 4357 | 939 | 3417405 |
| Max. Value | 1285 | 1051 | 1325 | 1198 | 1067 | 1011 | 1312 | 1090 | 1325 |
| Min. Value | 1333 | 1220 | 1390 | 1341 | 1185 | 1210 | 1480 | 1206 | 1185 |
|  | **FT (ºC)** | | | | | | | | |
|  | **RA** | **OA** | **TR** | **RI** | **CO** | **BA** | **SU** | **WH** | **TOT** |
| Average | 1310 | 1146 | 1363 | 1288 | 1148 | 1157 | 1411 | 1181 | 1250 |
| Median | 1308 | 1153 | 1373 | 1291 | 1162 | 1180 | 1450 | 1188 | 1239 |
| Standard desviation | 18 | 68 | 27 | 34 | 42 | 66 | 66 | 31 | 20 |
| Variance | 314 | 4632 | 733 | 1147 | 1793 | 4401 | 4357 | 939 | 3417405 |
| Max. Value | 1285 | 1051 | 1325 | 1198 | 1067 | 1011 | 1312 | 1090 | 1325 |
| Min. Value | 1333 | 1220 | 1390 | 1341 | 1185 | 1210 | 1480 | 1206 | 1185 |

**Table S6** ANOVA results of the proximate parameters of the straw crop residues. Rapeseed (RA), oats (OA), triticale (TR), rice (RI), corn (CO), barley (BA), sunflower (SU), and wheat (WH).

|  | **A_d (%)** | | | | |
| --- | --- | --- | --- | --- | --- |
|  | **Sum of Squares** | **Gl** | **Mean Square** | **F-Ratio** | **P-Value** |
| Between Groups | 1187.1 | 7 | 169.586 | 43.49 | 0 |
| Within Groups | 261.237 | 67 | 3.89906 |  |  |
| Total (Corr.) | 1448.34 | 74 |  |  |  |
|  | **V_d (%)** | | | | |
|  | **Sum of Squares** | **Gl** | **Mean Square** | **F-Ratio** | **P-Value** |
| Between Groups | 621.851 | 7 | 88.8359 | 12.21 | 0 |
| Within Groups | 487.555 | 67 | 7.27694 |  |  |
| Total (Corr.) | 1109.41 | 74 |  |  |  |
|  | **GCV (MJ/kg)** | | | | |
|  | **Sum of Squares** | **Gl** | **Mean Square** | **F-Ratio** | **P-Value** |
| Between Groups | 89.675 | 7 | 12.8107 | 8.69 | 0 |
| Within Groups | 98.8094 | 67 | 1.47477 |  |  |
| Total (Corr.) | 188.484 | 74 |  |  |  |
|  | **NCV (MJ/kg)** | | | | |
|  | **Sum of Squares** | **Gl** | **Mean Square** | **F-Ratio** | **P-Value** |
| Between Groups | 97.896 | 7 | 13.9851 | 9.54 | 0 |
| Within Groups | 98.2372 | 67 | 1.46623 |  |  |
| Total (Corr.) | 196.133 | 74 |  |  |  |

**Table S7.** ANOVA results of elementary analysis

|  | **C_d (%)** | | | | |
| --- | --- | --- | --- | --- | --- |
|  | **Sum of Squares** | **Gl** | **Mean Square** | **F-Ratio** | **P-Value** |
| Between Groups | 490.832 | 7 | 70.1188 | 9.48 | 0 |
| Within Groups | 495.408 | 67 | 7.39415 |  |  |
| Total (Corr.) | 986.239 | 74 |  |  |  |
|  | **H_d (%)** | | | | |
|  | **Sum of Squares** | **Gl** | **Mean Square** | **F-Ratio** | **P-Value** |
| Between Groups | 2.2328 | 7 | 0.318971 | 2.14 | 0.0515 |
| Within Groups | 10.0075 | 67 | 0.149366 |  |  |
| Total (Corr.) | 12.2403 | 74 |  |  |  |
|  | **N_d (%)** | | | | |
|  | **Sum of Squares** | **Gl** | **Mean Square** | **F-Ratio** | **P-Value** |
| Between Groups | 4.03325 | 7 | 0.576178 | 4.74 | 0.0002 |
| Within Groups | 8.02129 | 66 | 0.121535 |  |  |
| Total (Corr.) | 12.0545 | 73 |  |  |  |
|  | **S_d (%)** | | | | |
|  | **Sum of Squares** | **Gl** | **Mean Square** | **F-Ratio** | **P-Value** |
| Between Groups | 1.94462 | 7 | 0.277803 | 35.9 | 0 |
| Within Groups | 0.518443 | 67 | 0.00773796 |  |  |
| Total (Corr.) | 2.46307 | 74 |  |  |  |
|  | **O_d (%)** | | | | |
|  | **Sum of Squares** | **Gl** | **Mean Square** | **F-Ratio** | **P-Value** |
| Between Groups | 97.896 | 7 | 13.9851 | 9.54 | 0 |
| Within Groups | 98.2372 | 67 | 1.46623 |  |  |
| Total (Corr.) | 196.133 | 74 |  |  |  |
|  | **Cl_d (%)** | | | | |
|  | **Sum of Squares** | **Gl** | **Mean Square** | **F-Ratio** | **P-Value** |
| Between Groups | 5.6968 | 7 | 0.813828 | 29.14 | 0 |
| Within Groups | 1.87147 | 67 | 0.0279324 |  |  |
| Total (Corr.) | 7.56827 | 74 |  |  |  |

**Table S8.** ANOVA results of ash fusibility

|  | **DT (ºC)** | | | | |
| --- | --- | --- | --- | --- | --- |
|  | **Sum of Squares** | **Gl** | **Mean Square** | **F-Ratio** | **P-Value** |
| Between Groups | 815489 | 7 | 116498 | 25.34 | 0 |
| Within Groups | 307983 | 67 | 4596.76 |  |  |
| Total (Corr.) | 1.12E+06 | 74 |  |  |  |
|  | **ST (ºC)** | | | | |
|  | **Sum of Squares** | **Gl** | **Mean Square** | **F-Ratio** | **P-Value** |
| Between Groups | 540256 | 7 | 77179.4 | 29.22 | 0 |
| Within Groups | 176993 | 67 | 2641.69 |  |  |
| Total (Corr.) | 717249 | 74 |  |  |  |
|  | **HT (ºC)** | | | | |
|  | **Sum of Squares** | **Gl** | **Mean Square** | **F-Ratio** | **P-Value** |
| Between Groups | 602443 | 7 | 86063.3 | 40.15 | 0 |
| Within Groups | 143613 | 67 | 2143.48 |  |  |
| Total (Corr.) | 746056 | 74 |  |  |  |
|  | **FT (ºC)** | | | | |
|  | **Sum of Squares** | **Gl** | **Mean Square** | **F-Ratio** | **P-Value** |
| Between Groups | 602443 | 7 | 86063.3 | 40.15 | 0 |
| Within Groups | 143613 | 67 | 2143.48 |  |  |
| Total (Corr.) | 746056 | 74 |  |  |  |

**Table S9.** Moisture content, Mad. Rapeseed (RA), oats (OA), triticale (TR), rice (RI), corn (CO), barley (BA), sunflower (SU), and wheat (WH).

|  | **Moisture Content** |  | **Moisture Content** |
| --- | --- | --- | --- |
| **RA** | 6.20±0.140 | **CO** | 6.90±0.080 |
|  | 8.11±0.190 |  | 7.14±0.070 |
|  | 8.40±0.090 |  | 6.44±0.060 |
|  | 5.96±080 |  | 6.38±0.060 |
|  | 4.60±050 |  | 7.18±0.090 |
|  | 7.20±020 |  | 7.39±0.080 |
| **OA** | 7.46±0.014 | **BAR** | 8.11±0.050 |
|  | 8.05±0.080 |  | 8.01±0.080 |
|  | 6.89±0.110 |  | 7.99±0.060 |
|  | 7.19±0.050 |  | 8.05±0.100 |
|  | 8.25±0.040 |  | 7.55±0.090 |
|  | 8.40±0.110 |  | 7.14±0.070 |
| **TR** | 7.73±0.280 |  | 7.66±0.060 |
|  | 8.41±0.530 |  | 8.50±0.080 |
|  | 6.92±0.320 |  | 8.00±0.080 |
|  | 3.12±0.190 |  | 7.59±0.060 |
|  | 6.27±0.150 |  | 7.11±0.060 |
|  | 3.82±0.230 |  | 7.69±0.070 |
| **RI** | 8.10±0.170 | **SUN** | 9.50±0.130 |
|  | 6.66±0.080 |  | 9.95±0.110 |
|  | 7.42±0.090 |  | 10.01±0.100 |
|  | 7.41±0.080 |  | 9.44±0.090 |
|  | 7.41±0.050 |  | 10.66±0.080 |
|  | 7.95±0.090 |  | 9.69±0.070 |
|  | 7.36±0.060 |  | 10.50±0.120 |
|  | 7.61±0.080 | **WHE** | 8.55±0.090 |
|  | 6.90±0.060 |  | 9.14±0.090 |
|  | 6.64±0.090 |  | 8.89±0.070 |
|  | 7.04±0.040 |  | 9.25±0.080 |
|  | 7.19±0.040 |  | 9.85±0.110 |
|  | 6.95±0.080 |  | 8.88±0.100 |
|  | 7.12±0.070 |  | 9.55±0.090 |
|  | 7.11±0.070 |  | 9.90±0.060 |
|  | 7.16±0.060 |  | 8.75±0.050 |
|  | 7.18±0.040 |  | 9.70±0.060 |
|  | 6.87±0.090 |  | 8.40±0.080 |


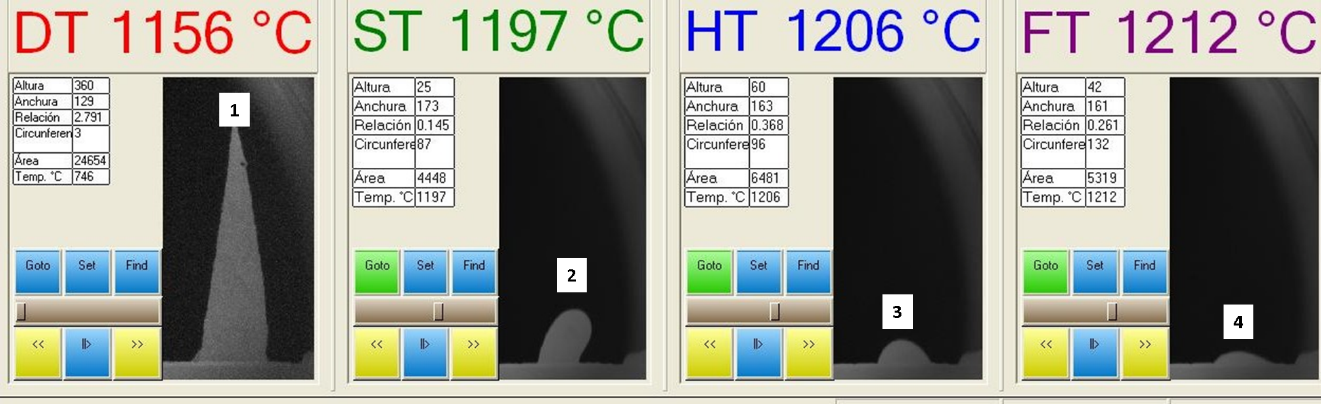


**Figure S1.** Example screenshot of the pyramidal ash pellet at different characteristic temperatures: (1) at DT temperature, (2) at ST, (3) at HT, and (4) at FT.
